# Prognostic Significance of CRP, BMI, and Ferritin Levels in Predicting COVID-19 Outcomes in Psychiatric Patients: A Comparative Analysis

**DOI:** 10.1192/j.eurpsy.2025.1264

**Published:** 2025-08-26

**Authors:** K. Argyropoulos, A.-A. Argyropoulou-Grizanou, P. Gourzis, E. Jelastopulu

**Affiliations:** 1 Public Health, School of Medicine, University of Patras, Patras; 2 Social Sciences, Hellenic Open University, Athens; 3 Psychiatry, School of Medicine, University of Patras, Patras, Greece

## Abstract

**Introduction:**

This study investigates the predictive value of ferritin, CRP, and BMI levels in predicting COVID-19 outcomes among psychiatric patients.

**Objectives:**

Understanding these markers is crucial given the increased vulnerability of psychiatric patients to severe COVID-19 outcomes.

**Methods:**

We conducted a retrospective analysis of 100 psychiatric patients diagnosed with COVID-19. We collected clinical outcomes (survival or death) data, demographic characteristics, and comorbidities. Blood samples were analyzed for ferritin and CRP levels, and BMI was calculated based on recorded weight and height. Statistical tests, including t-tests and chi-square tests, were used to assess the relationships between these variables and COVID-19 outcomes. Survival analysis was performed using the Log-Rank test to evaluate the impact of these markers on patient survival.

**Results:**

Our results showed that higher ferritin levels were significantly associated with poorer outcomes, with survivors having a mean ferritin level of 246.2 (SD = 150.3) compared to 416.9 (SD = 215.9) in non-survivors (p < 0.001). Similarly, mean CRP levels were lower in survivors (1.58, SD = 1.96) compared to non-survivors (3.46, SD = 2.92) with a p-value of 0.002. BMI did not show a statistically significant difference between survivors and non-survivors (p = 0.429). Survival analysis revealed that elevated CRP and ferritin levels correlated with decreased survival rates.

**Conclusions:**

The study highlights the significant role of ferritin and CRP as prognostic markers in psychiatric patients with COVID-19, suggesting that elevated levels of these biomarkers are associated with worse outcomes. However, BMI did not significantly affect survival, indicating that inflammatory markers might be more relevant for assessing prognosis in this population. These findings emphasize the need for vigilant monitoring of these biomarkers in psychiatric patients to manage their COVID-19 treatment better and improve outcomes.

**Disclosure of Interest:**

None Declared

